# Deep-reef fish assemblages of the Great Barrier Reef shelf-break (Australia)

**DOI:** 10.1038/s41598-017-11452-1

**Published:** 2017-09-07

**Authors:** Tiffany L. Sih, Mike Cappo, Michael Kingsford

**Affiliations:** 1 ARC Centre of Excellence for Coral Reef Studies and Marine Biology & Aquaculture, College of Science and Engineering, James Cook University, Townsville City, Australia; 2 AIMS@JCU partnership with Australian Institute of Marine Science, Townsville City, Australia; 3 Australian Institute of Marine Science, Townsville City, Australia

## Abstract

Tropical mesophotic and sub-mesophotic fish ecology is poorly understood despite increasing vulnerability of deeper fish assemblages. Worldwide there is greater fishing pressure on continental shelf-breaks and the effects of disturbances on deeper fish species have not yet been assessed. Difficult to access, deeper reefs host undocumented fish diversity and abundance. Baited Remote Underwater Video Stations (BRUVS) with lights were used to sample deeper habitats (54–260 m), in the Great Barrier Reef (GBR), Australia. Here we describe fish biodiversity, relative abundance and richness, assessing the prediction that depth would drive assemblage structure in the GBR. Distinct groups of fishes were found with depth whilst overall richness and abundance decreased steeply between 100 and 260 m. Commercially-valuable Lutjanidae species from *Pristipomoides* and *Etelis* genera, were absent from shallower depths. Few fish species overlapped between adjacent depth strata, indicating unique assemblages with depth. We also detected new location records and potential new species records. The high biodiversity of fish found in shelf-break environments is poorly appreciated and depth is a strong predictor of assemblage composition. This may pose a challenge for managers of commercial fisheries as distinct depth ranges of taxa may translate to more readily targeted habitats, and therefore, an inherent vulnerability to exploitation.

## Introduction

Fishes occupying deeper shelf-break environments are susceptible to increasing threats as the condition of many shallower coral reefs is in decline due to the effects of anthropogenic and environmental disturbances (*e.g*. fishing, pollution, coral bleaching and warming temperatures^1, 2^). Deeper mesophotic reefs are extensions of shallow habitats and can play a critical role in maintaining the health of the greater ecosystem^3^. Deeper environments may be refuges for shallow-reef fishes threatened by fishing pressure^4, 5^ and warming temperatures^6^. Worldwide, fishers are fishing deeper and more efficiently with better technology and gear^7–9^. The value of these ecosystems must be evaluated in the face of potential rapid future exploitation. What are critical – or irreplaceable – components to protect for future resources? Only by pushing the depth boundaries of ecological studies can we understand if deeper benthic habitats have similar or different patterns and processes. Further, to what degree are shallow and deep habitats connected? We need methods that can be used in both shallower and deeper habitats for comparisons over a broad geographic range.

There is a paucity of ecological information on the distribution and abundance of deep-reef fishes worldwide^10, 11^. The light-limited depths of the mesophotic and sub-mesophotic, which traditionally has remained a mystery due to the greater logistics^12^ and costs^11, 13^ of sampling deeper, and often remote, habitats. Mesophotic coral reefs can extend to 150 m in clear waters^14, 15^ and this depth is thought to be the lower distribution of many reef-based species^5, 16–18^, including fishes. Studies on mesophotic fish ecology may not sample the greater taxonomic diversity available^19^ because time, cost and expertise are often limited. However, deep-reefs may have a disproportionally high number of novel or endemic species^20–22^. The current information on deeper fish distribution is also not evenly distributed worldwide; it is currently unclear whether deep-reef fishes are found in broad geographic ranges but so far are only found in a few explored locations^11, 17, 20^.

The greatest proportion of reef fish biodiversity studies are limited to depths shallower than 30 m^13, 22^. This presents a large bathymetric gradient of reef communities that have not been explicitly described. Mesophotic fish and coral assemblages may change along depth gradients^13, 14, 22^ and may include shallower-occurring species, but also deep-specialist species restricted to certain depths^5, 17, 23–27^. The Great Barrier Reef (GBR) comprises 2,500 reefs and represents the world’s largest continuous coral reef ecosystem covering approximately 344,400 km^2^ ^28^. With over 1500 known fish species in the Great Barrier Reef Marine Park^29^ (GBRMP), few studies include the mesophotic depths along the edge of the continental shelf ^30^. This shelf-break may potentially have greater species diversity than mesophotic reefs in other study locations^22, 30, 31^ as follows: (1) the western Pacific and Australia is close to the “centre of reef biodiversity”^32–34^, (2) the broad shelf of the GBR harbours greater diversity^33^ and (3) the amount of deeper reef habitat may have been previously underestimated^35^. The continental shelf-edge can be among the steepest of environmental gradients, subject to a wide range of environmental drivers that can significantly change over tens of meters and affect the faunal diversity (*e.g*. light availability, temperature, benthic substrate, and food availability)^36^ and we predicted that there would be distinct fish communities along this gradient.

Depth is likely a key driver of assemblage structure^19, 25, 37, 38^ and evidence in the mesophotic so far concurs with this paradigm. Bathymetric breaks have been established for the GBR for coral species, including a transition at 60 m between distinct upper and lower mesophotic tropical assemblages^39^ and at subtropical latitudes around 50 m^40^. Fish species richness appears to increase to a maximum at 25–30 m, then decreases to 50–65 m^19^, however, these studies did not investigate deeper, to the maximum extent of these light-limited reef environments. Understanding how species richness is distributed across environmental gradients, such as the shallow-to-deep reef transition zone, is key to understanding how species in both zones may respond to future environmental changes. Further, bathymetric distribution data can improve conservation and management efforts and reduce bycatch, by encouraging fisheries to target depth ranges with a high proportion of target species relative to unwanted species.

Monitoring techniques often focus on economically important fishes, limiting the ability to detect changes in whole fish assemblages^41–43^. Underwater video has great potential to document and monitor deep-reef communities of fish and can be constructed to survey deeper depths with adequate light. Specifically, Baited Remote Underwater Videos Stations (BRUVS) have been used to monitor fish and benthic assemblages of the GBR, but not fish communities in deeper mesophotic and sub-mesophotic reef and inter-reefal habitats^37, 44^. BRUVS are useful for studying deep-reef fishes, as they can withstand pressures associated with greater depths and are easily replicated for repeatable ecological studies (see reviews^45–47^). Surveys with similar baited video equipment have assessed mesophotic fish communities in other locations, investigating abundance and size distributions^48–50^, habitat associations^49, 51^, and the efficacy of Marine Protected Areas for fisheries management^52, 53^. However, no studies have investigated below the 80 m isobath in the GBRMP^37^. BRUVS have inherent biases that have to be carefully considered, such as the presence of a bait plume, which can alter the behavior of fishes and preferentially attract larger, more mobile fishes (see reviews^45–47^). However, an advantage of this method is that it is not intrusive or destructive, thus BRUVS are permitted in most zones of the GBRMP. BRUVS are a good method in baseline and longterm deep-reef studies in the GBR as the images and video are geo-referenced and can be kept as a permanent record to validate fish identifications, or to compare species compositions over temporal and spatial scales with controlled sampling effort along a great depth range.

**Figure 1:**
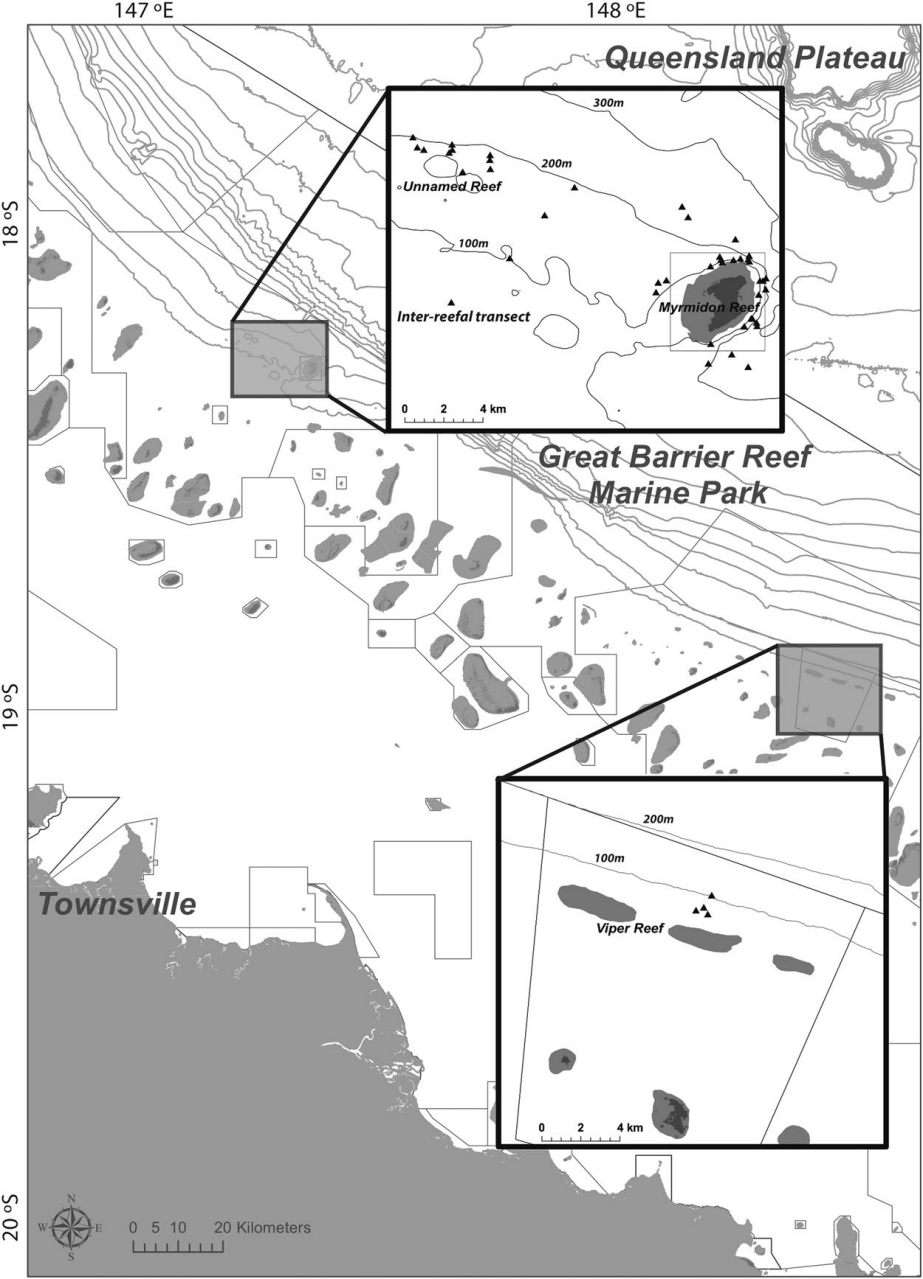
Map of Baited Remote Underwater Video Station surveys along the outer shelf-break of the Great Barrier Reef, Australia. Forty-eight BRUVS deployments (*triangles*) across three reefs (Unnamed, Myrmidon, and Viper) and an inter-reefal transect. The edge of the continental shelf is approximately 100 km offshore for the Central Great Barrier Reef. Map created in ArcMap 10.2.1 (http://desktop.arcgis.com/en/arcmap ^144^) with bathymetric contour lines (100 m) from Project 3DGBR (www.deepreef.org ^145^) and shapefiles provided by the Great Barrier Reef Marine Park Authority (http://www.gbrmpa.gov.au/resources-and-publications/spatial-data-information-services ^146^).

The objective of this study was to use BRUVS to investigate tropical fish assemblages in mesophotic to sub-mesophotic depths at a number of reefs along the shelf-edge of the central GBR (Fig. 1). We hypothesized that abundance of fishes and related diversity would vary with depth and that the patterns would be consistent by reef. This is the first comprehensive fishery-independent survey of mesophotic fish biodiversity within the GBR at depths of 50–300 m. Specifically, we aimed to: (a) determine how species richness and abundance vary with depth; (b) describe fish assemblages and identify key depth-indicator species; and (c) provide critical baseline information, which is archived for future comparisons; (d) measure thermal profiles of the water column, in multiple years where we hypothesized that temperature/depth strata may correlate with the distribution of fishes.

**Figure 2:**
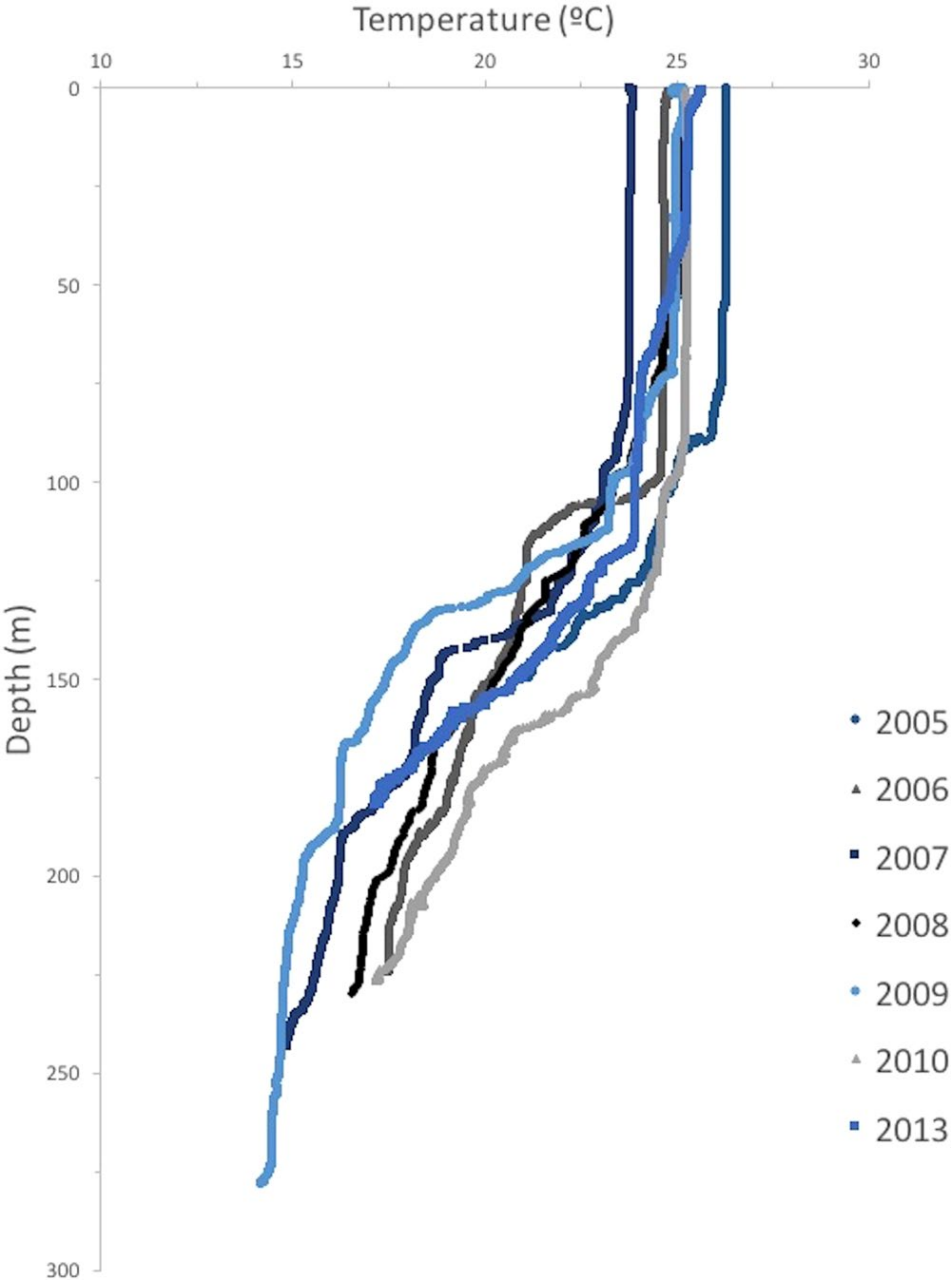
Position of the well-mixed layer and thermoclines in deep tropical waters off the shelf-break of the Great Barrier Reef, Australia. The data from 2005 to 2008 are redrawn from Walther *et al*.^95^.

Seawater temperature varied greatly with depth (Fig. 2). At Myrmidon, CTD data from 2009–2013 indicated surface temperatures were about 25 °C and well-mixed to approximately 100 m. Temperatures dropped by up to 10 °C (*i.e*. 14–16 °C) from ~100 m to a depth of ~250 m. The thermocline commenced at 70–100 m and in many years a decrease in temperature continued to the 200–250 m depth stratum with some evidence that the rate of change slowed at the greatest depths we sampled. Although the steepness of the temperature change at the beginning and within the thermocline varied among years, the depth of the well-mixed shallow water layer was similar from year to year.

## Results

A total of 1081 individual fish, sharks and rays were identified, representing 130 species from 29 families (48 BRUVS deployments, 42.35 hours of sampling-time). Species diversity varied with 1–40 species identified per deployment, average species richness was 9.44 species, and mean abundance of 22.5 fishes. Lutjanidae, Lethrinidae and Nemipteridae were the families most frequently sighted. The most speciose families were Labridae (23 spp), Carangidae (16 spp), Lutjanidae (16 spp), and Lethrinidae (11 spp). BRUVS allowed us to identify large-bodied fish such as groupers, jacks, snappers and apex predators such as sharks. Many commercially-valuable species were sighted including *Pristipomoides filamentosus*, *Pristipomoides multidens*, and *Plectropomus laevis*. Some smaller species and juveniles were only identified to genus (*i.e*. juvenile *Lethrinus sp*.).

Some of the species seen at these depths are of conservation concern according to IUCN criteria^54^, these include: Scalloped Hammerhead and Humphead Maori Wrasse (*Sphyrna lewini* and *Cheilinus undulatus*, Endangered), Blotched Fantail Ray, Silvertip Shark and Sandbar Shark (*Taeniurops meyeni*, *Carcharhinus albimarginatus* and *Carcharhinus plumbeus*, Vulnerable), and Whitetip Reef Shark and Grey Reef Shark (*Triaenodon obesus* and *Carcharhinus amblyrhynchos*, Near Threatened).

Several of the species observations represent new geographic location records for Australia, and specifically the GBR (Table 1). These include *Chromis okamurai* (143 m)^55^, *Chromis mirationis* (155–194 m)^56^, *Chromis circumaurea* (115 m)^20^ and the recently described *Bodianus bennetti* (155–179)^57^. Unrecognized species from *Selenanthias* (143–160 m), *Chromis* (155 m), and *Bodianus* (143 m) were also observed and may potentially be new species (Supplementary video).

**Table 1:** Fish species identified in deep-reef Baited Remote Underwater Video Station videos from the Central Great Barrier Reef shelf-break.

Species	CAAB code	Australian standard name	Depths observed (Number of videos)	Reported depth range	Depth extension?	Climate and known distribution	New record to the Great Barrier Reef or Australia
**Carcharhinidae**							
*Carcharhinus albimarginatus* (Rüppell, 1837)	37018027	Silvertip Shark	98–155 m (13)	1–800 m		Tropical Indo-Pacific	No
*Carcharhinus amblyrhynchos* (Bleeker, 1856)	37018030	Grey Reef Shark	54–156 m (10)	0–1000 m		Tropical Indo-West & Central Pacific	No
*Carcharhinus plumbeus* (Nardo, 1827)	37018007	Sandbar Shark	259 m (1)	0–500 m		Subtropical Atlantic & Indo-Pacific	No
*Loxodon macrorhinus* Müller & Henle, 1839	37018005	Sliteye Shark	107 m (1)	7–100 m	Marginal	Tropical Indo-West Pacific	No
*Triaenodon obesus* (Rüppell, 1837)	37018038	Whitetip Reef Shark	54–99.5 m (3)	1–330 m		Tropical Indo-Pacific	No
**Sphyrnidae**							
*Sphyrna lewini* (Griffith & Smith, 1834)	37019001	Scalloped Hammerhead	105 m (1)	0–1000 m		Circumglobal, tropical and temperate seas	No
**Dasyatidae**							
*Taeniurops meyeni* (Müller & Henle, 1841)	37035017	Blotched Fantail Ray	54 m (1)	1–500 m		Tropical Indo-West Pacific	No
**Muraenidae**							
*Gymnothorax berndti* Snyder, 1904	37060089	Y-Patterned Moray*	150 m (1)	30–303 m		West Indo-Pacific	Yes, new to GBR
*Gymnothorax elegans* Bliss, 1883	37060090	Elegant Moray*	110–149 m (2)	92–450 m		Indo-West Pacific	No, known from unpublished records
*Gymnothorax intesi* (Fourmanoir & Rivaton, 1979)	37060076	Whitetip Moray	200 m (1)	200–400 m		Subtropical West Pacific	No
*Gymnothorax prionodon* Ogilby, 1895	37060049	Sawtooth Moray	150–194 m (2)	20–80 m	Yes	Subtropical to temperate West Pacific	No
**Fistulariidae**							
*Fistularia commersonii* Rüppell, 1838	37278001	Smooth Flutemouth	54 m (1)	0–200 m		Tropical Indo-Pacific	No
**Peristediidae**							
*Satyrichthys* sp.	37288912		245 m (1)				
**Serranidae**							
*Epinephelus cyanopodus* (Richardson, 1846)	37311145	Purple Rockcod	99.5–102 m (2)	2–150 m		Tropical West Pacific	No
*Epinephelus morrhua* (Valenciennes, 1833)	37311151	Comet Grouper	115–194 m (6)	80–370 m		Tropical Indo-Pacific	No
*Plectranthias kelloggi* Jordan & Evermann, 1903	37311210	Eastern Flower Porgy*	155–179 m (2)	60–540 m		Temperate Pacific	Yes
*Plectropomus leopardus* (Lacépède, 1802)	37311078	Common Coral Trout	100–105 m (2)	3–100 m	Marginal	Tropical West Pacific	No
*Plectropomus laevis* (Lacépède, 1801)	37311079	Bluespotted Coral Trout	85–128 m (4)	4–100 m	Yes	Tropical Indo-Pacific	No
*Pseudanthias engelhardi* (Allen & Starck, 1982)	37311115	Barrier Reef Basslet	100 m (1)	37–70 m	Yes	Tropical West-Central Pacific	No
*Selenanthias* sp.	37311947		143–179 m (6)	129–204 m		Subtropical to temperate West Pacific	Yes, new to GBR
*Variola louti* (Forsskål, 1775)	37311166	Yellowedge Coronation Trout	54–98 m (2)	3–300 m		Tropical Indo-Pacific	No
**Malacanthidae**							
*Hoplolatilus marcosi* Burgess, 1978	37331012	Redback Sand Tilefish*	100 m (1)	18–80 m	Yes	Tropical Indo-Pacific	No
**Echeneidae**							
*Echeneis naucrates* Linnaeus, 1758	37336001	Sharksucker	54–155 m (8)	0–200 m	Yes	Subtropical; Circumtropical	No
**Carangidae**							
*Carangoides caeruleopinnatus* (Rüppell, 1830)	37337021	Onion Trevally	54–129 m (12)	1–60 m	Yes	Tropical Indo-West Pacific	No
*Carangoides chrysophrys* (Cuvier, 1833)	37337011	Longnose Trevally	54–60 m (2)	30–60 m		Indo-Pacific	No
*Carangoides dinema* Bleeker 1851	37337078	Shadow Trevally	54–102 m (4)	1–22 m	Yes	Tropical Indo-West Pacific	No
*Carangoides ferdau* (Forsskål, 1775)	37337068	Blue Trevally	57–100 m (2)	1–60 m	Yes	Tropical Indo-Pacific	No
*Carangoides fulvoguttatus* (Forsskål, 1775)	37337037	Turrum	99.5–102 m (2)	?–100m	Marginal	Indo-West Pacific	No
*Carangoides orthogrammus* (Jordan & Gilbert, 1882)	37337057	Thicklip Trevally	85–129 m (3)	3–168 m		Tropical Indo-Pacific	No
*Carangoides plagiotaenia* Bleeker, 1857	37337070	Barcheek Trevally	106 m (1)	2–200 m		Tropical Indo-Pacific	No
*Caranx ignobilis* (Forsskål, 1775)	37337027	Giant Trevally	54–85 m (2)	10–188 m		Tropical Indo-Pacific	No
*Caranx melampygus* Cuvier, 1833	37337050	Bluefin Trevally	54–85 m (2)	0–190 m		Tropical Indo-Pacific	No
*Decapterus* sp.	37337901		107–155 m (2)				
*Gnathanodon speciosus* (Forsskål, 1775)	37337012	Golden Trevally	102 m (1)	0–162 m		Tropical Pacific	No
*Pseudocaranx dentex* (Bloch & Schneider, 1801)	37337062	Silver Trevally	99.5–155 m (2)	10–238 m		Tropical Atlantic and Indo-Pacific	No
*Seriola dumerili* (Risso, 1810)	37337025	Amberjack	146–260 m (11)	1–360 m		Sub-tropical, circumglobal	No
*Seriola rivoliana* Valenciennes, 1833	37337052	Highfin Amberjack	98–245 m (10)	5–250 m		Sub-tropical, circumglobal	No
**Lutjanidae**							
*Aphareus rutilans* Cuvier, 1830	37346001	Rusty Jobfish	85–245 m (23)	10–330 m		Tropical Indo-Pacific	No
*Aprion virescens* Valenciennes, 1830	37346027	Green Jobfish	54–105 m (2)	0–180 m		Tropical Indo-Pacific	No
*Etelis carbunculus* Cuvier, 1828	37346014	Ruby Snapper	226 m (1)	90–400 m		Tropical Indo-Pacific	No
*Lipocheilus carnolabrum* (Chan, 1970)	37346031	Tang’s Snapper	194 m (1)	90–340 m		Indo-West Pacific	No
*Lutjanus bohar* (Forsskål, 1775)	37346029	Red Bass	85–128 m (10)	4–180 m		Tropical Indo-Pacific	No
*Lutjanus sebae* (Cuvier, 1816)	37346004	Red Emperor	99.5–103 m (2)	5–180 m		Tropical Indo-West Pacific	No
*Paracaesio kusakarii* Abe, 1960	37346060	Saddleback Snapper	156–200 m (3)	100–310 m		Tropical West Pacific	No
*Pristipomoides argyrogrammicus* (Valenciennes, 1831)	37346054	Ornate Jobfish	193–245 m (6)	70–350 m		Tropical Indo-Pacific	No
*Pristipomoides auricilla* (Jordan, Evermann & Tanaka, 1927)	37346059	Goldflag Snapper	150–194 m (3)	90–360 m		Indo-Pacific	No
*Pristipomoides filamentosus* (Valenciennes, 1830)	37346032	Rosy Snapper	85–201 m (16)	40–400 m		Indo-Pacific	No
*Pristipomoides multidens* (Day, 1870)	37346002	Goldbanded Snapper	129–250 m (14)	40–350 m		Tropical & sub-tropical Indo-Pacific	No
*Pristipomoides sieboldii* (Bleeker, 1857)	37346064	Lavender Snapper	143 m (1)	100–500 m		Indo-Pacific	No
*Pristipomoides typus* Bleeker, 1852	37346019	Sharptooth Snapper	115–250 m (18)	40–180 m	Yes	Tropical Indo-Pacific	No
*Symphorus nematophorus* (Bleeker, 1860)	37346017	Chinamanfish	60–105 m (4)	20–100 m	Marginal	Tropical West Pacific	No
**Caesionidae**							
*Pterocaesio marri* Schultz, 1953	37346068	Bigtail Fusilier	54 m (1)	1–35 m	Yes	Tropical Indo-Pacific	No
**Symphysanodontidae**							
*Symphysanodon* sp.	37346930		115 m (1)				
**Nemipteridae**							
*Nemipterus balinensis* (Bleeker, 1859)	37347039	Bali Threadfin Bream	194–240 m (2)	50–150 m	Yes	Tropical Indo-West Pacific	No
*Pentapodus aureofasciatus* Russell, 2001	37347029	Yellowstripe Threadfin Bream	54–106 m (7)	5–80 m	Yes	Tropical Pacific	No
*Pentapodus nagasakiensis* (Tanaka, 1915)	37347012	Japanese Threadfin Bream	100 m (1)	2–100 m		Tropical West Pacific	No
*Scolopsis* sp.	37347902		65 m (1)				
**Lethrinidae**							
*Gymnocranius euanus* (Günther, 1879)	37351022	Paddletail Seabream	54–156 m (10)	15–50 m	Yes	Tropical West Pacific	No
*Gymnocranius grandoculis* (Valenciennes, 1830)	37351005	Robinson’s Seabream	54–155 m (10)	20–170 m		Tropical Indo-Pacific	No
*Lethrinus laticaudis* Alleyne & Macleay, 1877	37351006	Grass Emperor	54 m (1)	5–35 m	Yes	Tropical West Pacific	No
*Lethrinus miniatus* (Forster, 1801)	37351009	Redthroat Emperor	54–128 m (8)	5–250 m		Tropical West Pacific	No
*Lethrinus nebulosus* (Forsskål, 1775)	37351008	Spangled Emperor	100–179 m (2)	0–90 m	Yes	Tropical Indo-West Pacific	No
*Lethrinus olivaceus* Valenciennes, 1830	37351004	Longnose Emperor	54–105 m (5)	1–185 m		Tropical Indo-West Pacific	No
*Lethrinus ravus* Carpenter & Randall, 2003	37351031	Drab Emperor	54–128 m (5)	5–35 m	Yes	Tropical West Pacific	No
*Lethrinus rubrioperculatus* Sato, 1978	37351012	Spotcheek Emperor	54–106 m (8)	8–198 m		Tropical Indo-Pacific	No
*Lethrinus semicinctus* Valenciennes, 1830	37351016	Blackblotch Emperor	54 m (1)	4–35 m	Yes	Tropical Indo-West Pacific	No
*Wattsia mossambica* (Smith, 1957)	37351027	Mozambique Seabream	105–160 m (8)	100–300 m		Tropical Indo-West Pacific	No
**Mullidae**							
*Mulloidichthys pfluegeri* (Steindachner, 1900)	37355040	Orange Goatfish	54–103 m (3)	13–200 m		Tropical Indo-West Pacific	Yes
*Parupeneus heptacantha* (Lacépède, 1802)	37355004	Cinnabar Goatfish	54–103 m (4)	12–350 m		Tropical Indo-West Pacific	No
*Parupeneus multifasciatus* (Quoy & Gaimard, 1825)	37355026	Banded Goatfish	54 m (1)	3–161 m		Tropical Pacific	No
*Parupeneus pleurostigma* (Bennett, 1831)	37355027	Sidespot Goatfish	100 m (1)	1–120 m		Tropical Indo-Pacific	No
**Chaetodontidae**							
*Heniochus diphreutes* Jordan, 1903	37365005	Schooling Bannerfish	128 m (1)	5–210 m		Subtropical Indo-Pacific	No
**Pomacanthidae**							
*Pomacanthus imperator* (Bloch, 1787)	37365014	Emperor Angelfish	100–105 m (2)	1–100 m		Tropical Indo-Pacific	No
*Pomacanthus semicirculatus* (Cuvier, 1831)	37365080	Blue Angelfish	105 m (1)	1–40 m	Yes	Tropical Indo-West Pacific	No
**Cirrhitidae**							
*Cyprinocirrhites polyactis* (Bleeker, 1875)	37374006	Lyretail Hawkfish	100 m (1)	10–132 m		Tropical Indo-West Pacific	No
**Pomacentridae**							
*Chromis circumaurea* Pyle, Earle & Greene, 2008	37372153	Gold-rim Chromis*	115 m (1)	?–100m	Yes	Tropical West Pacific	Yes
*Chromis mirationis* Tanaka 1917	37372048	Japanese Puller	155–194 m (2)	40–208 m		Subtropical West Pacific	Yes, new to GBR
*Chromis okamurai* Yamakawa & Randall, 1989	37372154	Okinawa Chromis*	143 m (1)	135–175 m		Subtropical to temperate Northwest Pacific	Yes
*Chromis* sp.	37372155		155 m (1)				Potential new species
**Labridae**							
*Bodianus anthioides* (Bennett, 1832)	37384052	Lyretail Pigfish	54 m (1)	6–60 m		Tropical Indo-Pacific	No
*Bodianus bimaculatus* Allen, 1973	37384055	Twospot Pigfish	100–106 m (2)	30–70 m	Yes	Tropical Indo-Pacific	No
*Bodianus izuensis* Araga & Yoshino, 1975	37384058	Striped Pigfish	98–105 m (2)	12–70 m	Yes	Subtropical West Pacific	Yes
*Bodianus masudai* Araga & Yoshino, 1975	37384221		115–155 m (2)	30–113 m	Yes	Subtropical: West Pacific anti-tropical distribution	Yes
*Bodianus bennetti*	37384219	Lemon-striped Pygmy Hogfish	155–179 m (4)	97–130 m	Yes	West Pacific	Yes, new to GBR, recently published record from the Coral Sea
*Bodianus* sp. 1	37384220		143 m (1)				Potential new species
*Cheilinus undulatus* Rüppell, 1835	37384038	Humphead Maori Wrasse	54 m (1)	1–100 m		Tropical Indo-Pacific	No
*Choerodon venustus* (De Vis, 1884)	37384042	Venus Tuskfish	54 m (1)	10–95 m		Subtropical West Pacific	No
*Cirrhilabrus punctatus* Randall & Kuiter, 1989	37384083	Finespot Wrasse	54–85 m (2)	2–78 m	Yes	Tropical West Pacific	No
*Cirrhilabrus roseafascia* Randall & Lubbock, 1982	37384218	Pink-Banded Fairy Wrasse*	85–155 m (8)	30–100 m	Yes	Tropical West Pacific	Yes, new to GBR, recently published record from the Coral Sea
*Cirrhilabrus* sp.	37384910		54–200 m (2)				
*Coris dorsomacula* Fowler, 1908	37384093	Pinklined Wrasse	60 m (1)	2–45 m	Yes	Tropical West Pacific	No
*Halichoeres* sp.	37384920		54 m (1)				
*Labroides dimidiatus* (Valenciennes, 1839)	37384028	Common Cleanerfish	54 m (1)	1–40 m	Yes	Tropical Indo-Pacific	No
*Labridae* sp.	37384000		54 m (1)				
*Oxycheilinus digrammus* (Lacépède, 1801)	37384065	Violetline Maori Wrasse	179–193 m (2)	3–120 m	Yes	Tropical Indo-Pacific	No
*Oxycheilinus orientalis* Günther, 1862	37384030	Oriental Maori Wrasse	99.5–110 m (2)	10–80 m	Yes	Tropical Indo-West Pacific	No
*Oxycheilinus* sp.	37384933		150 m (1)				
*Terelabrus rubrovittatus* Randall & Fourmanoir, 1998	37384210	Yellowbar Hogfish*	100–179 m (8)	50–140 m	Yes	Tropical Western Central Pacific; Japan; Maldives	Yes
**Pinguipedidae**							
*Parapercis nebulosa* (Quoy & Gaimard, 1825)	37390005	Pinkbanded Grubfish	105–179 m (11)	11–120 m	Yes	Tropical Indo-West Pacific	No
*Parapercis* sp.	37390901		60–245 m (10)				
**Blenniidae**							
*Meiacanthus luteus* Smith-Vaniz, 1987	37408054	Yellow Fangblenny	100 m (1)	0–40 m	Yes	Tropical West Pacific	No
**Acanthuridae**							
*Acanthurus xanthopterus* Valenciennes, 1835	37437020	Yellowmask Surgeonfish	100 m (1)	1–120 m		Tropical Indo-Pacific	No
*Naso caesius* Randall & Bell, 1992	37437046	Silverblotched Unicornfish	100–106 m (4)	15–50 m	Yes	Tropical Pacific	No
**Scombridae**							
*Gymnosarda unicolor* (Rüppell, 1836)	37441029	Dogtooth Tuna	85–260 m (17)	10–300 m		Tropical Indo-Pacific	No
*Scomberomorus commerson* (Lacépède, 1800)	37441007	Spanish Mackerel	54–155 m (4)	0–200 m		Tropical Indo-West Pacific	No
**Balistidae**							
*Abalistes stellatus* (Anonymous, 1798)	37465011	Starry Triggerfish	54–128 m (6)	7–350 m		Tropical Indo-West Pacific	No
*Balistidae* sp.	37465000		54 m (1)				
*Balistoides conspicillum* (Bloch & Schneider, 1801)	37465031	Clown Triggerfish	54–105 m (2)	1–75 m	Yes	Tropical Indo-Pacific	No
*Sufflamen bursa* (Bloch & Schneider, 1801)	37465078	Pallid Triggerfish	54 m (1)	3–90 m		Tropical Indo-Pacific	No
*Sufflamen fraenatum* (Latreille, 1804)	37465014	Bridled Triggerfish	98–107 m (4)	8–200 m		Tropical Indo-Pacific	No
**Tetraodontidae**							
*Torquigener* sp.	37467913		240 m (1)				
*Trionodon macropterus* Lesson, 1831	37991885	Threetooth Puffer*	245 m (1)	50–300 m		Tropical Indo-West Pacific	No

Identifications to species designation where possible and taxonomic information based on the Australian Faunal Directory^58^ and California Academy of Sciences’ Catalog of Fishes^59^. CAAB codes are the eight-digit Codes for Australian Aquatic Biota maintained by CSIRO Division of Marine and Atmospheric Research for species of research or commercial interest. Australian standard names are according to the Australian Faunal Directory or *FishBase^60^ common name. FishBase, Fishes of Australia^61^, IUCN Redlist^54^, Randall’s *Reef and Shore Fishes of the South Pacific* ^62^ and Allen and Erdmann’s *Reef Fishes of the East Indies* app^63^ were consulted for reported depth range. Where differences in these references occurred, the maximum depth range is reported. Climate and known distribution information from FishBase. New record information was compared to reported data from FishBase, Fishes of Australia and Atlas of Living Australia^64^ databases and cross-referenced with John Pogonoski (CSIRO).

A number of small-bodied fishes were recorded and are likely an underestimate of true abundance and richness. Both *Terelabrus rubriovittatus* and *Cirrhilabrus roseafascia* appeared in a large proportion (17%) of the sites. Other frequently sighted smaller fish include small *Bodianus* species (25% of sites) and *Pentapodus* species (19%).

### Species richness and abundance with depth

**Figure 3:**
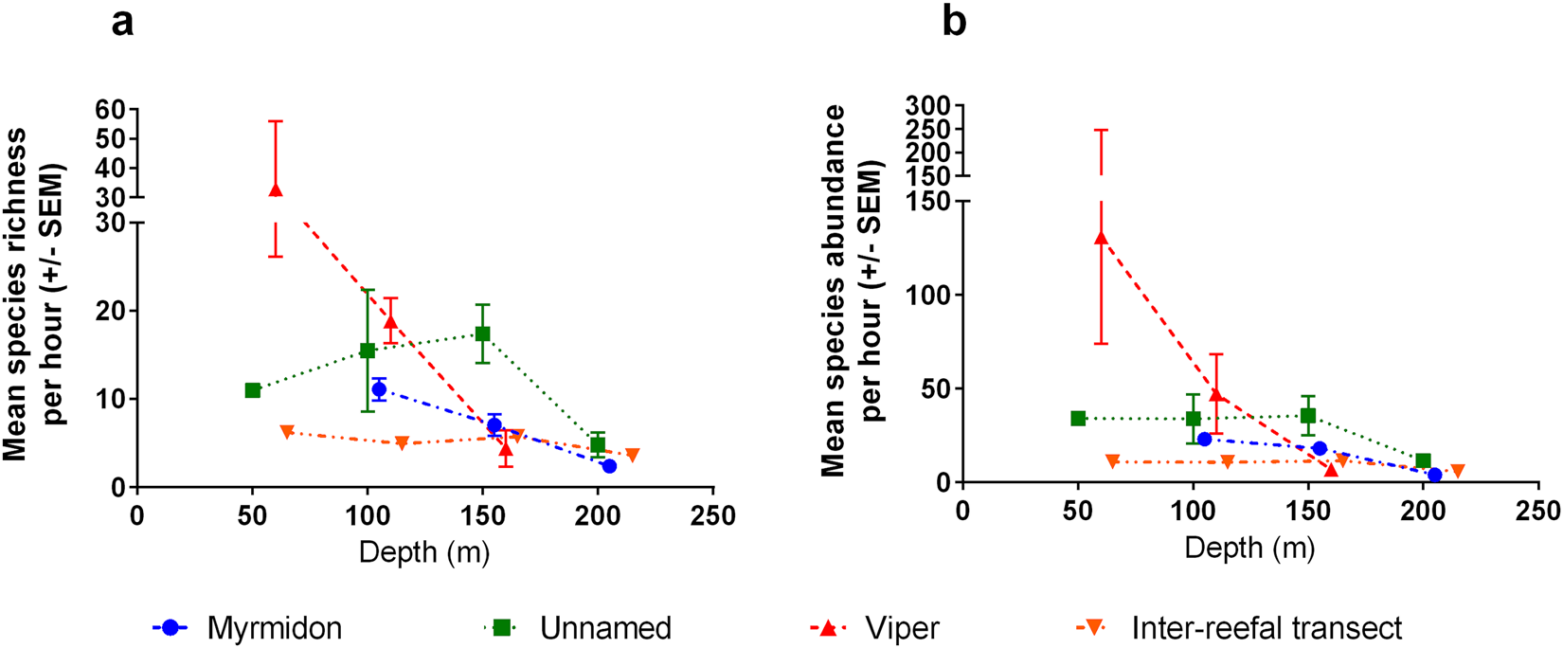
Species richness and abundance decline with increasing depth along the Great Barrier Reef shelf-break. (**a**) Mean total species richness and (**b**) Mean total species abundance (standardized per hour of sampling time). Symbols correspond to the three reefs and inter-reefal transect and are off-set for ease of interpretation. Sites were pooled into four depth strata: upper mesophotic (54–65 m, *n* = 4), middle mesophotic (85–115 m, *n* = 14), lower-mesophotic (128–160 m, *n* = 16), and sub-mesophotic (179–260 m, *n* = 15).

**Figure 4:**
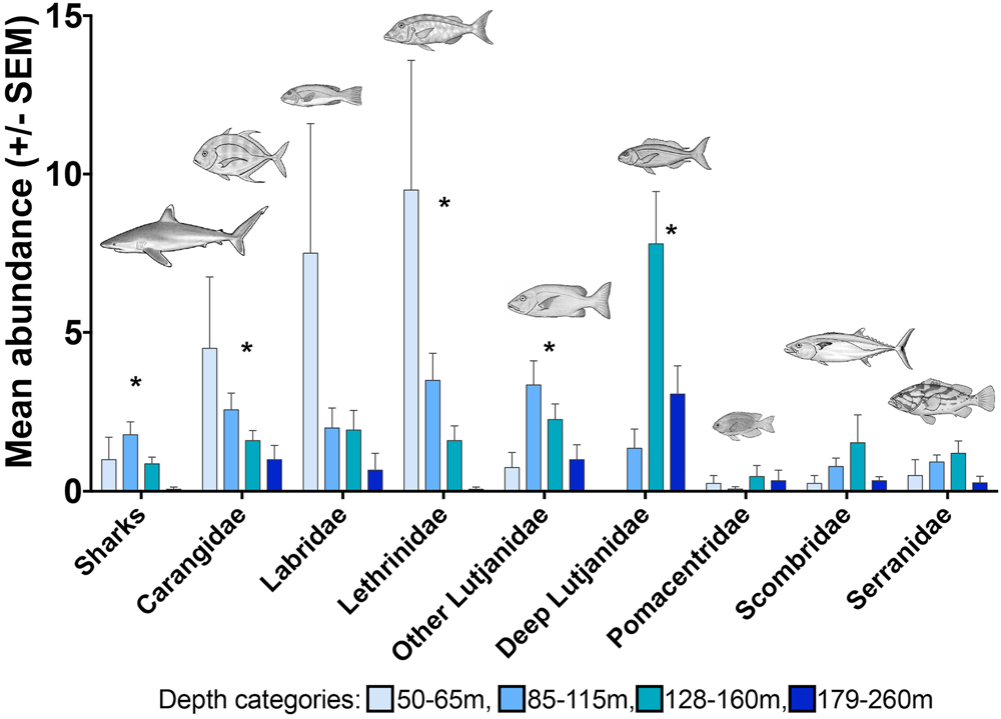
Mean total abundance of fish families sighted by Baited Remote Underwater Video Stations along the Great Barrier Reef shelf-break. Abundance was measured as MaxN per species per site, total abundance by family was the sum of all species relative abundance per site per depth category. Significantly different means (ANOVA) per depth are indicated by *. Illustrations drawn by Juliet Corley and copyright permission is granted by authors MC and TS.

Strong depth-related patterns of relative species richness (number of species per 60 minutes of video) and total fish abundance (sum of MaxN of all species per deployment per 60 minutes of video) were detected and these differences were significant according to ANOVA (Table 2). There was no interaction between depth and site (@ > p = 0.25) and therefore the interaction was pooled into the factor depth. Species richness and abundance generally decreased from shallow to deep although patterns varied by reef (Fig. 1). Comparing Shallow (50–115 m), Mid (128–160 m) and Deep (179–260 m) fish assemblage groups for species richness (t-tests), Shallow-Mid (*p* = 0.08, NS) and Mid-Deep (*p* = 0.06, NS) were not significantly different groups, but Shallow-Deep was (*p* = 0.02*). Tukey’s HSD highlighted the same differences in overall species richness between the depth groups: Shallow-Mid (p = 0.21, NS), Mid-Deep (p = 0.13, NS), and Shallow-Deep (p = 0.001*). Species abundance based on summed MaxN of all species present at each site showed a similar pattern, with non-significant differences Shallow-Mid (p = 0.47, NS) and Mid-Deep (p = 0.18, NS), and Shallow-Deep was a significant change (p = 0.004*) in pairwise t-tests. Post-hoc Tukey’s HSD Shallow-Mid (p = 0.33, NS), Mid-Deep (p = 0.14, NS) and Shallow-Deep (p = 0.004*). Variation of relative species abundance within depth strata was high, as indicated by standard error (SE) of 27–63% of the mean abundance per depth (Fig. 3). There was also variation in relative species richness within depths, SEs 19–49% mean richness. For both richness and abundance there was a general decrease in the variation between sites from shallow to deep (Fig. 3). However, the variation within strata was not great enough to obscure strong depth-related patterns. The decline in relative species abundance was mirrored in some families, with carangids, labrids and lethrinids decreasing in abundance with depth (Fig. 4). Lutjanidae exhibited depth-related zonation between species, with species *Lutjanus bohar* and *L. sebae* found at shallower depths and species from *Pristipomoides* and *Etelis* genera only in deeper depths. Lethrinidae species *Gymnocranius euanus*, *G. grandoculis* and *Wattsia mossambica* occurred at depths down to 150–160 m, other lethrinid species occurred in 128 m or shallower. Some fish species were only present at depths greater than 100 m (*i.e. Pristipomoides aureofasciatus*, *Wattsia mossambica*, *Lipocheilus carnolabrum*, *Paracaesio kusakarii*; Table 1).

**Table 2:** Species richness and abundance decreased with depth across all reefs pooled (one-way ANOVA).

	**Df**	**SS**	**MS**	**F-value**	**p**
**Richness**					
Among depths	2	12.55	6.28	7.19	0.002*
Within depths	39	34.04	0.87		
**Abundance**					
Among depths	2	38.62	19.31	5.88	0.006*
Within depths	39	128.13	3.29

### Fish assemblages

**Figure 5:**
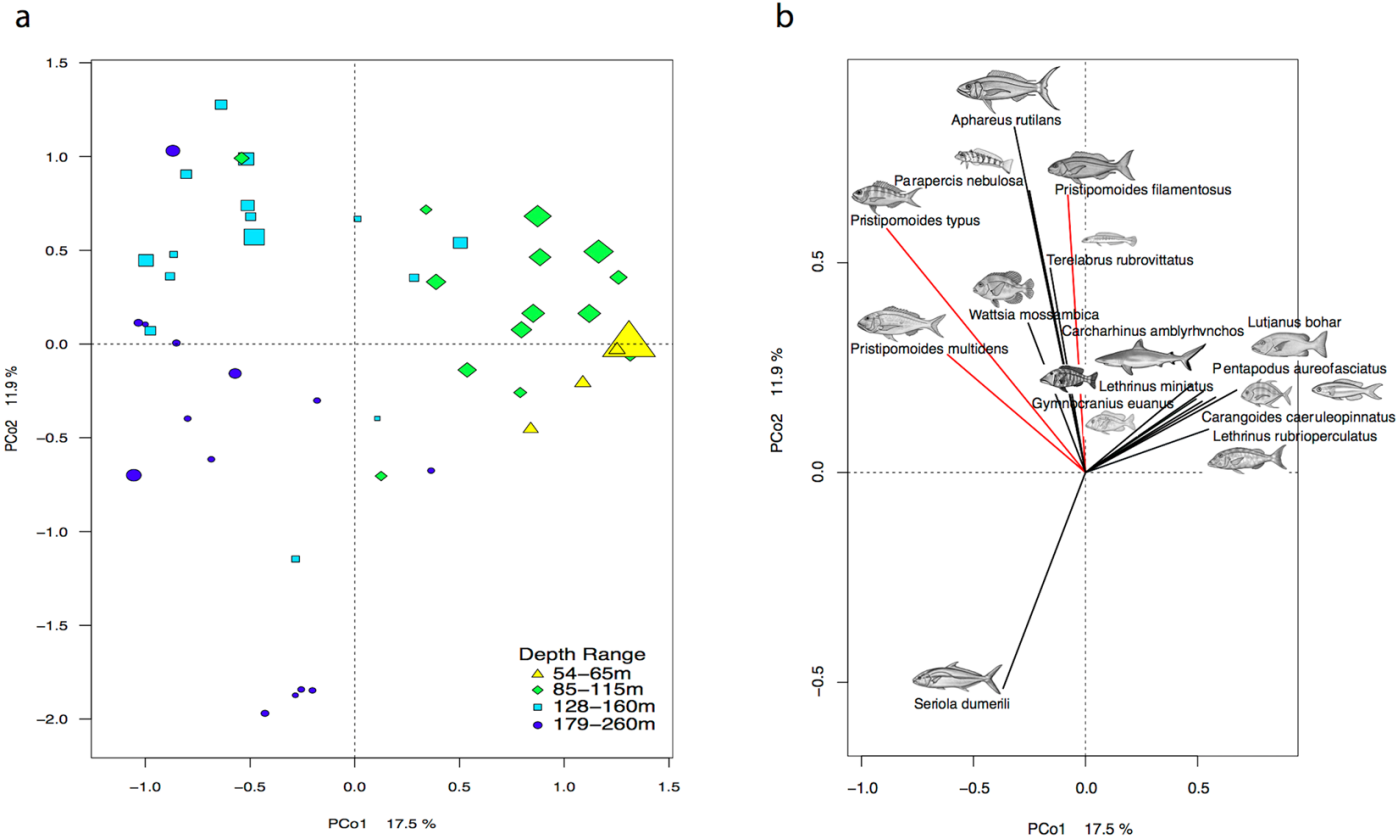
PCoA biplot of 47 Baited Remote Underwater Video Station sites: (**a**) Sites are color-coded by depth range and the size of the symbol corresponds to the total species richness scaled by a tenth; (**b**) 15 fish species scores are plotted that explain some of the variance between principle coordinates axes (scale of eigenvector is relative to the influence of that species to overall discrimination). Members of the *Pristipomoides* genus, prominent mesophotic fishes, are highlighted in red. Illustrations drawn by Juliet Corley and copyright permission is granted by authors MC and TS.

Fish assemblages varied with depth. PCo1 explained 17.5% of the variance and separated the deepest and shallowest sites (Fig. 5a). PCo2 separated the middle sites and explained 11.9% of the variance. Shallower sites (<100 m) were more speciose. *Seriola dumerili*, *Pristipomoides* species and the lethrinid *Wattsia mossambica* associated with deeper sites. *Lethrinus rubrioperculatus*, *Gymnocranius euanus*, *Pentapodus aureofasciatus*, and *Carangoides caeruleopinnatus* frequented shallower sites (Fig. 5b).

There was high species variation within depth strata and a number of single-species occurrences (*i.e*. species only recorded at one site). Fifty-eight species identified were only present in one site, resulting in high among-site diversity. Of single species occurrences, *MaxN* (the maximum number of a species within a single video frame) ranged from 1–85 individuals.

There were great differences in group membership by depth. However, in some cases there was species overlap in group memberships with depth (Table 3). Indicator species analysis of four pre-defined depth groups and multilevel pattern analysis attributed 130 species to a group or groups based on transformed species abundance. Twenty-three species were selected as having significant differences with depth: 13 were assigned to unique groups and ten species were assigned to two groups. No species were assigned to more than two groups. The upper mesophotic group (54–65 m) had a total of 36 unique species, of which seven were significantly attributed to only that depth strata (p < 0.05). The middle mesophotic group (85–115 m) was assigned 30 species with three significant. The lower mesophotic (128–160 m) had 18 species assigned, two were significant. The sub-mesophotic group (179–260 m) was assigned 13 species, only one was significant. There was a greater shared assemblage between the upper and middle mesophotic (11 species total), then between the upper and lower or the upper and sub-mesophotic groups. Middle and lower-mesophotic shared 11 species; the lower mesophotic and sub-mesophotic sites shared six species. The genus *Parapercis* (Family Pinguipedidae) was unusual in that it may be a depth-generalist genus, found in all three mesophotic groups (0.462, p = 0.765). Further, the highly mobile *Gymnosarda unicolor* (Family Scombridae) was found throughout the deepest groups (0.622, p = 0.363). Presence-absence data revealed almost identical results, out of 130 species 24 were selected: 12 were assigned to a unique group, 12 assigned to pairs of groups.

**Table 3:** Key fish indicator species per depth strata (multilevel pattern analysis).

	Upper mesophotic	Middle mesophotic	Lower mesophotic	Sub-mesophotic
	(54–65, n = 4)	(85–115 m, n = 14)	(128–160 m, n = 15)	(179–260 m, n = 15)
*Species which contribute significantly to each group*	*Abalistes stellatus* 0.957 *** a,o	*Lutjanus bohar* 0.774 ** a,o	*Pristipomoides typus* 0.760 ** a	*Pristipomoides argyrogrammicus* 0.632 ** a,o
	*Lethrinus rubrioperculatus* 0.752 ** a	*Sufflamen fraenatum* 0.535 * a,o	*Wattsia mossambica* 0.657 * a,o	
	*Lethrinus sp* 0.707 ** a,o	*Naso caesius* 0.535 * a,o	*Selenanthias sp* 0.449 * o	
	*Carangoides chrysophyrys* 0.707 ** a,o			
	*Mulloidichthys pfluegeri* 0.693 ** a,* o			
	*Gymnocranius grandoculis* 0.672 * a,o			
	*Carangoides dinema* 0.624 * a,o			
	**Group 1 + 2**	**Group 2 + 3**	**Group 3 + 4**	
*Species which contribute significantly to more than one group*	*Carangoides caeruleopinnatus* 0.756 ** a,o	*Aphareus rutilans* 0.756 ** a,o	*Pristipomoides multidens* 0.683 * a, ** o	
	*Lethrinus rubrioperculatus* 0.619 ** o	*Pristipomoides filamentosus* 0.679 * a,o	*Seriola dumerili* 0.606 * a,o	
	*Carcharinus amblyrhyncos* 0.691 ** a, * o	*Carcharinus albimarginatus* 0.670 * a, ** o	*Pristipomoides typus* 0.579 * o	
	*Gymnocranius euanus* 0.690 * a,o	*Cirrhilabrus roseafascia* 0.402 * o		
	*Pentapodus aureofasciatus* 0.624 * a,o			
	*Lethrinus miniatus* 0.611 * a			

IndVal index (0–1) is accompanied by significance levels: *p ≤ 0.05, **p ≤ 0.01, ***p ≤ 0.001; “a” for species abundance data, “o” for occurrence (presence-absence) data.

## Discussion

We found strong differences in fish assemblages with depth with high variability among reefs and sites within reefs. Further, we found distinct assemblages of fishes in mesophotic and sub-mesophotic habitats of the GBR, and these contrasted greatly with those of shallower shelf-habitats (*e.g. *soft bottom 20–90 m)^37, 65^, including those of coral reefs (<30 m)^66–68^. There are few comprehensive datasets on tropical deep-reef fishes, however, there is a growing body of comparable work in disparate locations, such as Hawaii, Brazil, Puerto Rico and the Caribbean. Our study is the first to characterize the diversity of deep-reef fish assemblages in the GBR. These depth patterns are similar to other deeper marine systems where the fish community shows strong zonation and declining species richness and abundance with the depth gradient (*e.g*. refs 19, 36, 49, 69–71). Some species show narrower depth ranges, while others are less restricted, and this has important implications for the future management of these resources. For instance, conservation planners can set aside representative areas based on depth to maximize protection of mesophotic reefs and species. Fishery managers can better define optimal targeted fishing depths and designate “Essential Fish Habitat” based on depth^72^, such as the designated Bottomfish Restricted Fishing Areas (BRFAs) implemented in the Hawaiian Bottomfish Fishery, for the protection of commercially important deep-reef fishes^48, 52, 53, 73, 74^.

Fisheries are vulnerable to the effects of fishing if there is limited habitat or constrained depth-ranges for target species. Shallow waters have been heavily impacted by fishing^75^. In the tropics, where the food security of many countries is uncertain, deeper reefs may be next in-line for greater fishing pressure. Many tropical coastlines that have limited shallower fishing areas are targeting deeper fisheries^76^. This is concerning as deeper environments are thought to be vulnerable^9, 76, 77^ and fish assemblages are poorly described^78, 79^, which may compound the problem. In general, deeper fish assemblages are thought to be diverse, valuable and vulnerable^80^. Since many of these species only occur at deeper depths, it is critical to consider these depth zones as distinct. Bycatch is one of the immeasurable impacts of fishing, therefore, it is important to inventory the biodiversity and value we may lose when we target deeper fisheries. High single-species occurrences can indicate the relative rarity of the fish taxa, but this can only be answered with future sampling and greater spatial replication. It is imperative, therefore, to obtain thorough baseline information on deeper tropical ecosystems before these species and habitats are compromised.

Some of the key indicator species per depth strata were commercially important species. Deep Lutjanidae (snappers from the genera *Aphareus*, *Etelis* and *Pristipomoides*), serranids, carangids and sharks are among the “largely unexplored fauna” of the Townsville area and continental slope^81^, and important for “regional food futures”^81^. Australia shares fauna with the south-western Pacific islands and the larger Indo-Pacific region^30^. As human populations increase across Australia and Indo-Pacific islands nations, pressure will be added to fish stocks throughout the region and sustainable fisheries management will increasingly become a major international political issue^81–83^.

In many Pacific nations, there are long-standing and emerging deep bottomfish fisheries and there is growing concern that these data-limited fisheries are vulnerable to the effects of overfishing^77, 84, 85^. In Hawaii, deep-reef lutjanids, serranids and carangids form the second largest fishery behind the tuna fishery^48^. For the majority of these fishes, biological information is lacking, but limited life history information demonstrate overall low production (see review^86^). “Essential Fish Habitat” has been set aside to reduce the impacts from fishing in Hawaiian waters^74^ and in other countries where these species are targeted similar precautionary measures should be made.

In Australia, deep-reef fishes are targeted by multiple methods along an extensive tropical coastline spanning Queensland, Western Australia and the Northern Territory. In the Northern Territory and Western Australia, mixed gear is used to target *Pristipomoides* species, primarily *Pristipomoides multidens* ^87, 88^, however, often multiple species are marketed under the same common name “Goldband snapper”^89^. In Western Australian waters deepwater demersal trawl gear is also used to target deep-reef fishes^90^. Fishing methods which target >50 species in ~200 m depths unfortunately catch many species as bycatch. In Queensland, while fishing pressure in deeper habitats of the GBR is comparatively lower than in shallow waters, more comprehensive information on deeper habitats will help to extend conservation strategies for the GBR World Heritage Area^35, 91^ and the adjacent Coral Sea^79, 81^ to incorporate deeper habitats.

Variation in fish assemblages was strongly correlated with depth and a combination of biotic and abiotic environmental conditions may contribute to this pattern. The thermocline and changing temperature and productivity with depth, may correlate with food for planktivores and piscivores^92, 93^. Position of the thermocline is probably a key factor driving the distribution of fishes^5, 94^. Our CTD data indicate temperatures rapidly decline below 100 m to 150–200 m, and similar profiles have been previously recorded in this area^95^; a steeper change than recorded in other tropical mesophotic regions^3, 5, 94^. This depth corresponds with a transition from the lower mesophotic assemblage to the sub-mesophotic fishes. Variation in physical properties (*i.e*. nutrients, light, oxygen and temperature) of the water column along with position and intensity of the thermocline influence species abundances in shallow tropical waters^96, 97^ and this appears to apply in deep shelf-waters^98^. Competition is also a powerful influence on species richness and abundance in shallower waters^99–101^ and more research on mesophotic competitive interactions is needed.

We found strong patterns of fish abundance with depth, but there was also some variation among reefs that may reflect depth-related patterns of habitat structural complexity^102–104^. Decreases or changes in fish diversity within depth strata may be linked to differences in available habitat similar to shallow water environments^36, 105–108^. Environmental drivers, such as currents and thermal stratification, will affect physical characteristics of the environment (*i.e*. temperature, sedimentation and food availability), which influence abundance and species diversity^109^. These abiotic factors affect the benthic community (the biotic structures, *e.g*. hard coral), which combined with the geomorphology, constitutes the habitat available to fishes^110^. Our results indicate inter-reefal habitats had lower relative species richness than those neighbouring reefs, suggesting the importance of the habitat type on diversity. Habitat quality may also explain some variation in relative species richness and abundance among reefs sampled.

Of the information necessary for conservation strategies, worldwide current species inventories and distributions are incomplete^111^. Further, data-poor locations inhibit the ability to monitor and record range extensions and distributional records. Analogous to the tropicalization of temperate waters^112, 113^, shallower species may extend their range and begin to inhabit deeper depths^114^. There is little information on how thermal tolerances may change fish distributions or behavior, such as changing spawning locations or moving deeper to avoid warm waters^6^. Distributional records and documented range extensions can be used as a “canary in a coalmine”; fishes as sentinel species can indicate the relative health of the broader ecosystems.

Shelf-break environments may be priority conservation hotspots, with high proportions of endemics^21, 22^ or species with restricted depth-ranges^33, 115^. Australia has high total endemism and up to a third of its demersal fishes may be endemic^30^, therefore, there may also be high endemism in its demersal shelf-break fish assemblages. We may also be underestimating the Australian shelf-break’s conservation value, as key bioregions including the upper continental slope of Queensland and the inter-reefal areas of the GBR are missing comprehensive fish assemblage information^31^. As genetic tools are increasing the resolution of cryptic speciation, there are likely differences detected between eastern and western Australian populations, and within species-complexes from neighboring regions^30^. Even without this information, Last *et al*. (2005, 2011) concluded that Australia-wide there were strong depth zonation patterns with characteristic and distinct demersal fish assemblages below 40 m. However, there was a “disjunction” at the shelf-edge between the continental shelf and slope bathomes assemblages (>40 m and <200 m), possibly due to “edge effects near the shelf break”^31^. We hypothesize that further investigation of shelf-edge habitats will demonstrate high diversity and distinctive communities. Shelf-break habitats should be considered intrinsically unique and a source of unforetold biodiversity and value.

There has been a rapid proliferation of reporting new species and new geographic records from mesophotic regions (*e.g*. refs 5, 20, 21, 25–27, 116–124). Even though underwater video cannot collect taxonomic samples^125, 126^, it can be a useful method for identifying hotspots for conservation priorities^32^. There were species we were unable to identify. While these represent a small percentage (<5%) of fish species identified from BRUVS deployments, the observations indicate there are other new species at depths previously unrecorded in the GBR. In our study, fish identifications can be scrutinized as images are listed by CAAB (Codes for Australian Aquatic Biota) codes in the AIMS database for future re-assessments of the identifications.

In conclusion, we found that depth was a strong predictor of fish assemblages at mesophotic and sub-mesophotic depths of the GBR. Our findings on the GBR align with other tropical and sub-tropical studies in deeper habitats. Distinct fish assemblages and high species diversity was found along the depth gradient and this potentially contributes to high levels of endemism in Australian fishes and other parts of the world. These narrow depth distributions may constitute an inherent vulnerability to targeted fishing pressures and should be incorporated in future regional management strategies.

## Materials and Methods

### BRUVS deployment

Three reefs were sampled along the shelf-edge (Myrmidon, Unnamed and Viper) and one inter-reefal transect using a depth-stratified sampling design (Fig. 1). Two identical BRUVS units rated to 300 m were used, with an aluminum elliptical roll-bar frame enclosing a camera-housing with a flat acrylic front port and battery-powered spotlight (white) mounted above the top roll-bar. Sony high-definition Handicams HDR-CX110 were used, with focus set to manual infinity to maximize the field of view. Using a bridle-rope configuration with twice the water depth of attached line per deployment, each BRUVS was marked by surface floats and flags for retrieval. The bait arm consisted of a plastic conduit to a plastic mesh bag filled with ~1 kg of crushed pilchards (*Sardinops sagax*, see review for the effect of bait^127, 128^).

Forty-eight deployments were made in May, June and Sept 2014 on three cruises. All deployments were placed during daylight (50–300 m of water depth; 0700–1800) with most of the effort targeting 100–300 m in transects at each reef with three targeted depth strata. Our hypothesis was that there would be differences in the fish assemblage with depth. BRUVS were deployed in shallow (~100 m), mid (~150 m) and deep (~200 m) strata at each reef. Viper Reef is on a shallower sloping shelf-edge, so depths of >200 m were not available without travelling substantially further offshore. Instead, BRUVS were deployed shallower to get a similar bathymetric depth gradient (50–150 m) over a similar spacing between deployments (*i.e*. differences would be due to depth, not increased distance from shore). Within depth-strata BRUVs were haphazardly-spaced several hundred meters apart.

### Fish identification and analysis of video metrics

Underwater imagery was read using Australian Institute of Marine Science (AIMS) purpose-built software. The following details were noted: time on the sea-bed, time of first appearance of each species, and abundance *N* of each species until time *MaxN* (highest number of individuals of a species per frame) reached, until the end of sampling (when the video left the bottom or when the tape finished recording). *MaxN* is a conservative estimate of abundance to eliminate the possibility of re-counting fish swimming in and out of the field-of-view^65^. Videos were read to its full length (27 to 84 minutes, average soak of 54 minutes) and later standardized for length of time of sampling (number of species present-absent per site for species richness, and number of fish per species for relative abundance, per 60 minute increment). Fish were identified to lowest possible taxa, with the assistance of fish experts, fish identification books and Fishbase.org ^60^. Every effort was made to identify large, conspicuous fish in addition to smaller, cryptic species. Fish identification photographs and BRUVS deployment metadata are archived in the Australian Institute of Marine Science database and can be accessed by request.

### Depth patterns

Species were summed across all sites for species richness and abundance. Where standardized values of total abundance and richness were used, the estimates were standardized by number of species per 60 minutes of sampling time. For our analyses two depth classification systems were used. For the one-way ANOVA which required a balanced design, three depth categories “Shallow” (50–115 m), “Mid” (128–160 m) and “Deep” (179–260 m) were used. For other analyses “Shallow” was further divided to two smaller categories to investigate the differences 50–115 m. Our sites were categorized in four depth strata: “upper mesophotic” (50–65 m), “middle mesophotic” (85–115 m), “lower mesophotic” (128–160 m) and “sub-mesophotic” (179–260 m). These strata represented breaks in the depth-stratified sampling design, but also aligned with previously documented transitional boundaries, including the ~150 m lower depth-limit of Mesophotic Coral Ecosystems (MCEs)^129^. Analyses were performed using several packages in R statistical software^130^ (CRAN ver. 3.2.3) and Excel.

To evaluate the general trend of how species richness and abundance varied with depth, standardized richness and abundance were square-root transformed and data were tested for any significant deviation from normality (Shapiro-Wilks: species richness Wilks = 0.98, p = 0.66; abundance Wilks = 0.95, p = 0.07) to meet the assumptions of ANOVA. In our original design we had the factors ‘Depth’ (*a* = 3) and ‘Reef’ (*b* = 3; Myrmidon, Unnamed, Viper) and site (*n* = 4) with an interaction between depth and site. The interaction was weak (*p* < 0.25), therefore, the factors were pooled as recommended by Underwood (1997)^131^. The factor ‘Reef’ was pooled for a stronger test for the factor ‘Depth’. ANOVA was performed for Depth (*a* = 3, *n* = 14) for both richness and abundance and two-tailed t-tests between depth groups with a Bonferroni correction was applied.

Mean standardized richness and abundance were also plotted in relation to depth strata separately by reef (Myrmidon, Viper and Unnamed; varied number of replicates within stratum). In addition, deployments were made along an inter-reefal transect (60–200 m, one replicate per depth). Shallower BRUVS sets from Viper Reef, one from on top of the submerged unnamed deep reef and the inter-reefal transect were included as an additional (50–65 m depth strata, *n* = 4). For analysis of separate families, we separated the Lutjanidae family into “deep” members (*Etelis* and *Pristipomoides* genera) and “other” (all other member species). Family analyses followed the one-way ANOVA for species richness and abundance.

### Investigating fish assemblages

We also wanted to investigate species associations as they may be better predictors of environmental conditions than species individually. This is often difficult because of positively-skewed frequency distributions and the high frequency of zeros in larger community composition datasets^132^. Species abundances (summed *MaxN*, maximum number of fish per species per site) were fourth-root transformed, which down-weights highly abundant species and reduces the skew in the distribution for each species^133^.

We used a Principle Coordinate Analysis (PCoA) ordination to visualise the differences between sites. Eliminating single-species occurrences (species only occurring at one site) from this analysis (58 of 130 species), we used 47 of the sites with 72 of the fish species in a Bray-Curtis dissimilarity matrix (packages vegan^134^, ecodist^135^). Agglomerative heirarchical unconstrained clustering revealed 12 significant clusters (SIMPROF; packages cluster^136^, clustersig^137^). For the ordination we color-coded the sites with the depth strata from the previous constrained univariate analyses and size-coded the symbols to correspond with species richness in the resulting biplot (functions capscale, vegdist). Capscale revealed ordination distances that were analogous to the original dissimilarities and is similar to redundancy analysis but can utilise non-Euclidean dissimilarities^134^. To determine which fish species corresponded with the variance between sites, we plotted the 15 species with the highest species scores.

We used species abundance data to perform multilevel pattern analysis of species by depth (functions multipatt, package indicspecies^138^). This method first lists species associated with particular groups of sites and then indicator species analysis is independently conducted for each species^139^. This method requires multiple testing, but can help to predict the likelihood of individual species to attribute to that depth assemblage^139^. Statistical significance is interpreted based on the IndVal index, which is a measure of association between the species and that depth group and tested through a permutation test^140^. An advantage of the function multipatt is that it looks for both indicator species for individual depth strata as well as combinations of strata^139^. We also repeated this analysis using presence-absence (occurrence) data using Pearson’s phi coefficient of association, a measure of the correlation used between binary variables (values of 0 and 1)^133^. Because this analysis is independently conducted for each species, we chose to include all species. Further, rare or single-species occurrences can be important for ecosystem functioning^141, 142^. We considered the inclusion of all species to align with our objective of describing complete assemblages, and rare species (*sensu* FishBase) are of higher conservation concern as they can be more sensitive to ecosystem stresses than common species^143^.

### Measurements of temperature with depth

On the outer shelf-edge off Myrmidon Reef, near the 300 m isopleth (Fig. 1), a *Seabird* Conductivity Temperature and Depth recording device was slowly lowered (<1 m/sec) by hand to an estimated maximum depth before retrieval. The instrument was calibrated for 60 seconds below the surface before deployment. Repeated samples were made in early August 2009, 2010 and 2013.

All methods in this study were carried out in accordance with local guidelines and regulations for the GBRMP. Experimental protocols were approved by the animal ethics committee at James Cook University. Methods were non-invasive and no animals were taken in this fieldwork.

### Data availability statement

BRUVS deployment information, recorded species and linked images are available by request from the Australian Institute of Marine Science. Map bathymetric contour lines from Dr. Rob Beaman and Project 3DGBR (www.deepreef.org); map shapefiles provided by the Great Barrier Reef Marine Park Authority (http://www.gbrmpa.gov.au/resources-and-publications/spatial-data-information-services).

## Electronic supplementary material

Caught on camera

Supplementary material

## References

[1] Hoegh-Guldberg O (1999). Climate change, coral bleaching and the future of the world’s coral reefs. Marine and Freshwater Research.

[2] Hughes TP (2003). Climate change, human impacts, and the resilience of coral reefs. Science.

[3] Lesser MP, Slattery M, Leichter JJ (2009). Ecology of mesophotic coral reefs. Journal of Experimental Marine Biology and Ecology.

[4] Lindfield SJ, Harvey ES, Halford AR, McIlwain JL (2016). Mesophotic depths as refuge areas for fishery-targeted species on coral reefs. Coral Reefs.

[5] Feitoza BM, Rosa RS, Rocha LA (2005). Ecology and zoogeography of deep-reef fishes in northeastern Brazil. Bulletin of Marine Science.

[6] Currey LM, Heupel MR, Simpfendorfer CA, Williams AJ (2015). Assessing environmental correlates of fish movement on a coral reef. Coral Reefs.

[7] Morato T, Watson R, Pitcher TJ, Pauly D (2006). Fishing down the deep. Fish and Fisheries.

[8] Roberts CM (2002). Deep impact: the rising toll of fishing in the deep sea. Trends in Ecology & Evolution.

[9] Cheung WW, Watson R, Morato T, Pitcher TJ, Pauly D (2007). Intrinsic vulnerability in the global fish catch. Marine Ecology Progress Series.

[10] Pyle, R. L. Use of advanced mixed-gas diving technology to explore the coral reef “Twilight Zone”. *Ocean Pulse: A Critical Diagnosis*, 71–88, doi:10.1007/978-1-4899-0136-1_9 (1998).

[11] Pyle RL (2000). Assessing undiscovered fish biodiversity on deep coral reefs using advanced self-contained diving technology. Marine Technology Society Journal.

[12] Gage, J. D. & Tyler, P. A. *Deep-sea biology: a natural history of organisms at the deep-sea floor* (Cambridge University Press, 1991).

[13] Kahng S (2010). Community ecology of mesophotic coral reef ecosystems. Coral Reefs.

[14] Kahng SE, Copus JM, Wagner D (2014). Recent advances in the ecology of mesophotic coral ecosystems (MCEs). Current opinion in environmental sustainability.

[15] Hinderstein LM (2010). Theme section on “Mesophotic Coral Ecosystems: Characterization, Ecology, and Management”. Coral Reefs.

[16] Colin PL (1974). Observation and collection of deep-reef fishes off the coasts of Jamaica and British Honduras (Belize). Marine Biology.

[17] Brokovich E, Einbinder S, Shashar N, Kiflawi M, Kark S (2008). Descending to the twilight-zone: changes in coral reef fish assemblages along a depth gradient down to 65 m. Mar Ecol Prog Ser.

[18] Garcia-Sais JR (2010). Reef habitats and associated sessile-benthic and fish assemblages across a euphotic-mesophotic depth gradient in Isla Desecheo, Puerto Rico. Coral Reefs.

[19] Pearson R, Stevens T (2015). Distinct cross-shelf gradient in mesophotic reef fish assemblages in subtropical eastern Australia. Marine Ecology Progress Series.

[20] Pyle RL, Earle JL, Greene BD (2008). Five new species of the damselfish genus *Chromis* (Perciformes: Labroidei: Pomacentridae) from deep coral reefs in the tropical western Pacific. Zootaxa.

[21] Last PR, Pogonoski JJ, Gledhill DC, White WT, Walker CJ (2014). The deepwater demersal ichthyofauna of the western Coral Sea. Zootaxa.

[22] Kane C, Kosaki RK, Wagner D (2014). High levels of mesophotic reef fish endemism in the Northwestern Hawaiian Islands. Bulletin of Marine Science.

[23] Bejarano I, Appeldoorn RS, Nemeth M (2014). Fishes associated with mesophotic coral ecosystems in La Parguera, Puerto Rico. Coral Reefs.

[24] Rosa MR (2016). Mesophotic reef fish assemblages of the remote St. Peter and St. Paul’s Archipelago, Mid-Atlantic Ridge, Brazil. Coral Reefs.

[25] Baldwin CC, Robertson DR (2014). A new *Liopropoma* sea bass (Serranidae, Epinephelinae, Liopropomini) from deep reefs off Curaçao, southern Caribbean, with comments on depth distributions of western Atlantic liopropomins. ZooKeys.

[26] Tornabene L, Robertson DR, Baldwin CC (2016). *Varicus lacerta*, a new species of goby (Teleostei, Gobiidae, Gobiosomatini, *Nes* subgroup) from a mesophotic reef in the southern Caribbean. ZooKeys.

[27] Baldwin CC, Robertson DR (2015). A new, mesophotic *Coryphopterus* goby (Teleostei, Gobiidae) from the southern Caribbean, with comments on relationships and depth distributions within the genus. ZooKeys.

[28] Great Barrier Reef Marine Park Authority. *Facts about the Great Barrier Reef*, http://www.gbrmpa.gov.au/about-the-reef/facts-about-the-great-barrier-reef (2016).

[29] Choat, J. & Russell, B. In *The Great Barrier Reef: Biology, environment and management*. (eds P. Hutchings, M. J. Kingsford, & O. Hoegh-Guldberg) 327-342 (CSIRO Publishing, 2008).

[30] Last PR (2011). Biogeographical structure and affinities of the marine demersal ichthyofauna of Australia. Journal of Biogeography.

[31] Last, P. R. *et al*. Validation of national demersal fish datasets for the regionalisation of the Australian continental slope and outer shelf (>40 m depth). Report No. 1876996870, (National Oceans Office, 2005).

[32] Allen, G. Indo-Pacific coral-reef fishes as indicators of conservation hotspots In *Proceedings of the Ninth International Coral Reef Symposium, Bali*, 23–27 *October 2000*. Vol. 2: 921–926 (2002).

[33] Allen GR (2008). Conservation hotspots of biodiversity and endemism for Indo-Pacific coral reef fishes. Aquatic Conservation: Marine and Freshwater Ecosystems.

[34] Bellwood DR, Hughes TP (2001). Regional-scale assembly rules and biodiversity of coral reefs. Science.

[35] Harris PT (2013). Submerged banks in the Great Barrier Reef, Australia, greatly increase available coral reef habitat. ICES Journal of Marine Science: Journal du Conseil.

[36] Zintzen, V. *et al*. Diversity and composition of demersal fishes along a depth gradient assessed by baited remote underwater stereo-video. *PLoS ONE* **7**, doi:10.1371/journal.pone.0048522 (2012). 10.1371/journal.pone.0048522 PMC3485343 23119045

[37] Cappo M, De’ath G, Speare P (2007). Inter-reef vertebrate communities of the Great Barrier Reef Marine Park determined by baited remote underwater video stations. Marine Ecology Progress Series.

[38] Gaston KJ (2000). Global patterns in biodiversity. Nature.

[39] Bridge TCL (2011). Variability in mesophotic coral reef communities along the Great Barrier Reef, Australia. Marine Ecology Progress Series.

[40] Malcolm HA, Jordan A, Smith SDA (2010). Biogeographical and cross-shelf patterns of reef fish assemblages in a transition zone. Marine Biodiversity.

[41] Magurran AE (2010). Long-term datasets in biodiversity research and monitoring: assessing change in ecological communities through time. Trends in Ecology & Evolution.

[42] Maxwell D, Jennings S (2005). Power of monitoring programmes to detect decline and recovery of rare and vulnerable fish. Journal of Applied Ecology.

[43] Depczynski M, Bellwood DR (2003). The role of cryptobenthic reef fishes in coral reef trophodynamics. Marine Ecology Progress Series.

[44] Cappo, M., Alongi, D. M., Williams, D. M. & Duke, N. A review and synthesis of Australian fisheries habitat research: Major threats, issues and gaps in knowledge of coastal and marine fisheries habitats. Report No. 0642322007, (Australian Institute of Marine Science, 1998).

[45] Mallet D, Pelletier D (2014). Underwater video techniques for observing coastal marine biodiversity: a review of sixty years of publications (1952–2012). Fish Res..

[46] Murphy HM, Jenkins GP (2010). Observational methods used in marine spatial monitoring of fishes and associated habitats: a review. Marine and Freshwater Research.

[47] Harvey, E. *et al*. The use of BRUVs as a tool for assessing marine fisheries and ecosystems: a review of the hurdles and potential. 2011 National Workshop, Project No. 2010/002 (2013).

[48] Moore CH, Drazen JC, Kelley CD, Misa W (2013). Deepwater marine protected areas of the main Hawaiian Islands: establishing baselines for commercially valuable bottomfish populations. Marine Ecology Progress Series.

[49] Fitzpatrick BM, Harvey ES, Heyward AJ, Twiggs EJ, Colquhoun J (2012). Habitat specialization in tropical continental shelf demersal fish assemblages. PLoS ONE.

[50] Merritt D (2011). BotCam: a baited camera system for nonextractive monitoring of bottomfish species. Fishery Bulletin.

[51] Misa WFXE, Drazen JC, Kelley CD, Moriwake VN (2013). Establishing species-habitat associations for four eteline snappers using a baited stereo-video camera system. Fishery Bulletin.

[52] Sackett D (2013). Marine protected areas for deepwater fish populations: an evaluation of their effects in Hawai’i. Marine Biology.

[53] Moore C, Drazen JC, Radford BT, Kelley C, Newman SJ (2016). Improving essential fish habitat designation to support sustainable ecosystem-based fisheries management. Marine Policy.

[54] IUCN 2016. *The IUCN Red List of Threatened Species*. Version 2016-3. http://www.iucnredlist.org (2016).

[55] Yamakawa T, Randall JE (1989). *Chromis okamurai*, a new damselfish from the Okinawa Trough, Japan. Japanese Journal of Ichthyology.

[56] Tanaka S (1917). Eleven new species of fish from Japan. Dobutsugaku Zasshi.

[57] Gomon MF, Walsh F (2016). A new pygmy hogfish (Labridae: *Bodianus*) of the subgenus Trochopus from the tropical southern Pacific Ocean. Journal of the Ocean Science Foundation.

[58] Australian Biological Resources Study (ABRS) 2009. Australian Faunal Directory. http://www.environment.gov.au/biodiversity/abrs/online-resources/fauna/afd/index.html (2009).

[59] Eschmeyer, W. N., Fricke, R. and van der Laan, R. (eds) *Catalog of fishes*. http://researcharchive.calacademy.org/research/ichthyology/catalog/fishcatmain.asp (2016).

[60] Froese, R. and Pauly, D. (eds) *FishBase*. http://www.fishbase.org (2016).

[61] Museums Victoria. *Fishes of Australia*. http://www.fishesofaustralia.net.au (2016).

[62] Randall, J. E. *Reef and shore fishes of the South Pacific: New Caledonia to Tahiti and the Pitcairn Islands*. Vol. 1 (University of Hawaii Press Honolulu, 2005).

[63] Allen, G. R. & Erdmann, M. V. *Reef fishes of theEast Indies* Vol. I-III (Tropical Reef Research Perth, Apple App Store, 2013).

[64] *Atlas of Living Australia*. http://www.ala.org.au (2016).

[65] Cappo, M. *Development of a baited video technqiue and spatial models to explain patterns of fish biodiversity in inter-reef waters* PhD thesis, James Cook University (2010).

[66] Williams DM (1982). Patterns in the distribution of fish communities across the central Great Barrier Reef. Coral Reefs.

[67] Russ GR (1989). Distribution and abundance of coral reef fishes in the Sumilon Island reserve, central Philippines, after nine years of protection from fishing. Asian Mar. Biol.

[68] Alevizon W, Richardson R, Pitts P, Serviss G (1985). Coral zonation and patterns of community structure in Bahamian reef fishes. Bulletin of Marine Science.

[69] Thresher RE, Colin PL (1986). Trophic structure, diversity and abundance of fishes of the deep reef (30–300 m) at Enewetak, Marshall Islands. Bulletin of Marine Science.

[70] Olavo G, Costa PA, Martins AS, Ferreira BP (2011). Shelf-edge reefs as priority areas for conservation of reef fish diversity in the tropical Atlantic. Aquatic Conservation: Marine and Freshwater Ecosystems.

[71] Pinheiro HT (2016). Upper and lower mesophotic coral reef fish communities evaluated by underwater visual censuses in two Caribbean locations. Coral Reefs.

[72] Rosenberg A, Bigford TE, Leathery S, Hill RL, Bickers K (2000). Ecosystem approaches to fishery management through essential fish habitat. Bulletin of Marine Science.

[73] Moore CH (2016). Improving spatial prioritisation for remote marine regions: optimising biodiversity conservation and sustainable development trade-offs. Scientific Reports.

[74] Kelley C, Moffitt R, Smith JR (2006). Mega- to micro-scale classification and description of bottomfish essential fish habitat on four banks in the Northwestern Hawaiian Islands. Atoll Research Bulletin.

[75] Jennings S, Polunin NV (1996). Impacts of fishing on tropical reef ecosystems. Ambio.

[76] Crossland, J. & Grandperrin, R. *The development of deep bottom fishing in the tropical Pacific* (South Pacific Commission, 1980).

[77] Fry GC, Brewer DT, Venables WN (2006). Vulnerability of deepwater demersal fishes to commercial fishing: Evidence from a study around a tropical volcanic seamount in Papua New Guinea. Fish Res..

[78] Hughes T, Connell J (1999). Multiple stressors on coral reefs: a long-term perspective. Limnology and oceanography.

[79] Bridge TC, Hughes TP, Guinotte JM, Bongaerts P (2013). Call to protect all coral reefs. Nature Climate Change.

[80] McKinnon AD (2014). Tropical marginal seas: Priority regions for managing marine biodiversity and ecosystem function. Annual Review of Marine Science.

[81] Young JW (2011). Workshop on the ecosystem and fisheries of the Coral Sea: an Australian perspective on research and management. Rev. Fish. Biol. Fish..

[82] Sainsbury, K., Campbell, R. & Whitelaw, A. Effects of trawling on the marine habitat on the north west shelf of Australia and implications for sustainable fisheries management. *Sustainable Fisheries Through Sustaining Fish Habitat. Canberra, Australia, Australian Government Publishing Service*, 137–145 (1993).

[83] Garcia SM (1994). The Precautionary Principle: its implications in capture fisheries management. Ocean & Coastal Management.

[84] Williams AJ (2013). Population biology and vulnerability to fishing of deep-water eteline snappers. Journal of Applied Ichthyology.

[85] Williams A (2012). International workshop on developing strategies for monitoring data-limited deepwater demersal line fisheries in the Pacific Ocean. Rev. Fish. Biol. Fish..

[86] Newman, S. J. *et al*. Review of the life history characteristics, ecology and fisheries for deep-water tropical demersal fish in the Indo-Pacific region. *Rev. Fish. Biol. Fish*. 1–26, doi:10.1007/s11160-016-9442-1 (2016).

[87] Lloyd, J., Ovenden, J. R., Newman, S. J. & Keenan, C. *Stock structure of Pristipomoides multidens resources across Northern Australia*. (Fisheries Research and Development Corporation, Fisheries Western Australia, Department of Primary Industry and Fisheries, Department of Primary Industries, Queensland Government, 1996).

[88] Newman SJ, Steckis RA, Edmonds JS, Lloyd J (2000). Stock structure of the goldband snapper *Pristipomoides multidens* (Pisces: Lutjanidae) from the waters of northern and western Australia by stable isotope ratio analysis of sagittal otolith carbonate. Marine Ecology Progress Series.

[89] Lloyd, J. A. *Summary of information on Goldband snapper* (*Pristipomoides spp*.) in the Northern Territory Dropline and Trap Fishery. 16 (Department of Primary Industry and Fisheries, Northern Territory of Australia, 2005).

[90] Rodgers, M., Sampaklis, A. & Pham, T. Western deepwater trawl fishery in *Fishery status reports 2009* Ch. 19, 337–349 (2010).

[91] Bridge TCL, Grech AM, Pressey RL (2016). Factors influencing incidental representation of previously unknown conservation features in marine protected areas. Conservation Biology.

[92] Paffenhöfer G-A (1983). Vertical zooplankton distribution on the northeastern Florida shelf and its relation to temperature and food abundance. Journal of Plankton Research.

[93] Barber RT, Chavez FP (1983). Biological consequences of El Niño. Science.

[94] Bongaerts P, Ridgway T, Sampayo EM, Hoegh-Guldberg O (2010). Assessing the ‘deep reef refugia’ hypothesis: focus on Caribbean reefs. Coral Reefs.

[95] Walther, B. D., Kingsford, M. J. & McCulloch, M. T. Environmental records from Great Barrier Reef corals: Inshore versus offshore drivers. *PLoS One* **8**, doi:10.1371/journal.pone.0077091 (2013). 10.1371/journal.pone.0077091 PMC3799737 24204743

[96] Hixon MA, Beets JP (1993). Predation, prey refuges, and the structure of coral reef fish assemblages. Ecological Monographs.

[97] Orth RJ, Heck KL, van Montfrans J (1984). Faunal communities in seagrass beds: a review of the influence of plant structure and prey characteristics on predator-prey relationships. Estuaries.

[98] Sainsbury K (1988). The ecological basis of multispecies fisheries and management of a demersal fishery in tropical Australia. Fish Population Dynamics.

[99] Holbrook SJ, Schmitt RJ (2002). Competition for shelter space causes density-dependent predation mortality in damselfishes. Ecology.

[100] Robertson DR (1996). Interspecific competition controls abundance and habitat use of territorial Caribbean damselfishes. Ecology.

[101] Robertson DR, Gaines SD (1986). Interference competition structures habitat use in a local assemblage of coral reef surgeonfishes. Ecology.

[102] Bridge TC (2011). Variability in mesophotic coral reef communities along the Great Barrier Reef, Australia. Mar Ecol Prog Ser.

[103] Bridge T (2011). Topography, substratum and benthic macrofaunal relationships on a tropical mesophotic shelf margin, central Great Barrier Reef, Australia. Coral Reefs.

[104] Amado-Filho GM (2016). Mesophotic ecosystems of the unique South Atlantic atoll are composed by rhodolith beds and scattered consolidated reefs. Marine Biodiversity.

[105] Suthers IM (2011). The strengthening East Australian Current, its eddies and biological effects - an introduction and overview. Deep Sea Research Part II: Topical Studies in Oceanography.

[106] Crowder LB, Cooper WE (1982). Habitat structural complexity and the interaction between bluegills and their prey. Ecology.

[107] Friedlander AM, Parrish JD (1998). Habitat characteristics affecting fish assemblages on a Hawaiian coral reef. Journal of Experimental Marine Biology and Ecology.

[108] Gratwicke B, Speight M (2005). The relationship between fish species richness, abundance and habitat complexity in a range of shallow tropical marine habitats. Journal of Fish Biology.

[109] Garrabou J, Ballesteros E, Zabala M (2002). Structure and dynamics of north-western Mediterranean rocky benthic communities along a depth gradient. Estuarine, Coastal and Shelf Science.

[110] Heyns ER, Bernard ATF, Richoux NB, Götz A (2016). Depth-related distribution patterns of subtidal macrobenthos in a well-established marine protected area. Marine Biology.

[111] Schultz AL, Malcolm HA, Bucher DJ, Linklater M, Smith SD (2014). Depth and medium-scale spatial processes influence fish assemblage structure of unconsolidated habitats in a subtropical marine park. PloS One.

[112] Vergés A (2014). The tropicalization of temperate marine ecosystems: climate-mediated changes in herbivory and community phase shifts. Proc. R. Soc. B.

[113] Last PR (2011). Long-term shifts in abundance and distribution of a temperate fish fauna: a response to climate change and fishing practices. Global Ecology and Biogeography.

[114] Munday PL, Jones GP, Pratchett MS, Williams AJ (2008). Climate change and the future for coral reef fishes. Fish and Fisheries.

[115] Roberts CM (2002). Marine biodiversity hotspots and conservation priorities for tropical reefs. Science.

[116] White WT (2011). *Odontanthias randalli* n. sp., a new anthiine fish (Serranidae: Anthiinae) from Indonesia. Zootaxa.

[117] Okamoto M, Motomura H (2012). *Epigonus exodon*, a new species of deepwater cardinalfish (Teleostei: Perciformes: Epigonidae) from Reunion, western Indian Ocean. Zootaxa.

[118] Allen GR, Erdmann MV (2009). Two new species of damselfishes (Pomacentridae: Chromis) from Indonesia. Aqua, International Journal of Ichthyology.

[119] Randall JE, Heemstra PC (2008). *Meganthias fìliferus*, a new species of anthiine fish (Perciformes: Serranidae), from the Andaman Sea off southwestern Thailand. Phuket Marine Biological Center Research Bulletin.

[120] Allen GR, Walsh F (2015). *Plectranthias bennetti*, a new species of anthiine fish (Pisces: Serranidae) from the Coral Sea, Australia. Journal of the Ocean Science Foundation.

[121] Baldwin CC, Robertson DR (2013). A new *Haptoclinus* blenny (Teleostei, Labrisomidae) from deep reefs off Curaçao, southern Caribbean, with comments on relationships of the genus. ZooKeys.

[122] Baldwin CC, Pitassy DE, Robertson DR (2016). A new deep-reef scorpionfish (Teleostei, Scorpaenidae, *Scorpaenodes*) from the southern Caribbean with comments on depth distributions and relationships of western Atlantic members of the genus. ZooKeys.

[123] Tornabene L (2016). Molecular phylogeny, analysis of character evolution, and submersible collections enable a new classification of a diverse group of gobies (Teleostei: Gobiidae: *Nes* subgroup), including nine new species and four new genera. Zoological Journal of the Linnean Society.

[124] Baldwin CC, Johnson GD (2014). Connectivity across the Caribbean Sea: DNA barcoding and morphology unite an enigmatic fish larva from the Florida Straits with a new species of sea bass from deep reefs off Curaçao. PloS one.

[125] Rocha LA (2014). Specimen collection: An essential tool. Science.

[126] Bello G, Causse R, Loipej L, Dulčić J (2014). A proposed best practice approach to overcome unverified and unverifiable “first records” in ichthyology. Cybium.

[127] Hardinge J, Harvey ES, Saunders BJ, Newman SJ (2013). A little bait goes a long way: the influence of bait quantity on a temperate fish assemblage sampled using stereo-BRUVs. Journal of Experimental Marine Biology and Ecology.

[128] Westerberg H, Westerberg K (2011). Properties of odour plumes from natural baits. Fish Res..

[129] Kahng SE (2010). Community ecology of mesophotic coral reef ecosystems. Coral Reefs.

[130] R Core Team. *R: A language and environment for statistical computing*. https://www.R-project.org/ (R Foundation for Statistical Computing, Vienna, Austria, 2015).

[131] Underwood, A. J. *Experiments in ecology: their logical design and interpretation using analysis of variance* (Cambridge University Press, 1997).

[132] Legendre P (2005). Species associations: the Kendall coefficient of concordance revisited. Journal of Agricultural, Biological, and Environmental Statistics.

[133] Borcard, D., Gillet, F. & Legendre, P. *Numerical ecology with R* (Springer Science & Business Media, 2011).

[134] Oksanen, J. *et al*. Package ‘vegan’. *Community ecology package, version* **2** (2013).

[135] Goslee SC, Urban DL (2007). The ecodist package for dissimilarity-based analysis of ecological data. Journal of Statistical Software.

[136] Maechler, M., Rousseeuw, P., Struyf, A., Hubert, M. & Hornik, K. Cluster: Cluster analysis and basics and extensions. https://cran.r-project.org/web/packages/cluster/index.html (2015).

[137] Whitaker, D. & Christman, M. Clustsig: Significant Cluster Analysis. *R package version 1.1* https://cran.r-project.org/package=clustsig (2014).

[138] De Cáceres M, Legendre P (2009). Associations between species and groups of sites: indices and statistical inference. Ecology.

[139] De Cáceres, M. How to use the indicspecies package (ver. 1.7.1). https://cran.r-project.org/web/packages/indicspecies/vignettes/indicspeciesTutorial.pdf (2013).

[140] Dufrêne M, Legendre P (1997). Species assemblages and indicator species: The need for a flexible asymmetrical approach. Ecological Monographs.

[141] Poos MS, Jackson DA (2012). Addressing the removal of rare species in multivariate bioassessments: the impact of methodological choices. Ecological Indicators.

[142] Lyons KG, Brigham C, Traut B, Schwartz MW (2005). Rare species and ecosystem functioning. Conservation Biology.

[143] Cao Y, Williams DD, Williams NE (1998). How important are rare species in aquatic community ecology and bioassessment?. Oceanography.

[144] ESRI. ArcGIS Desktop: Release 10.2.1, Redlands, CA: Environmental Systems Research Institute http://desktop.arcgis.com/en/arcmap (2014).

[145] Beaman, Rob. Great Barrier Reef and Coral Sea bathymetry. Deep Reef Exporer website. http://www.deepreef.org/ (2014).

[146] Great Barrier Reef Marine Park Authority, Great Barrier Reef Marine Park Zoning [dataset]. Identifier: EC12E1A4-36AE-4D5A-AB53-89D662FDF34E. Retrieved from: http://www.gbrmpa.gov.au/resources-and-publications/spatial-data-information-services (2014).

