# Supplementary material for: Deep-reef fish assemblages of the Great Barrier Reef shelf-break (Australia)
Source: Sci Rep. 2017 Sep 7;7:10886. doi: 10.1038/s41598-017-11452-1 (PMC5589835; doi:10.1038/s41598-017-11452-1)
Supplement: Supplementary file 2 — Supplementary material [file 41598_2017_11452_MOESM2_ESM.doc]

# Deep-reef fish assemblages of the Great Barrier Reef shelf-break (Australia)

Tiffany L. Sih^1,2^, Mike Cappo^3^, Michael Kingsford^1^

^1^ ARC Centre of Excellence for Coral Reef Studies and Marine Biology & Aquaculture, College of Science and Engineering,

James Cook University

^2^ AIMS@JCU partnership with Australian Institute of Marine Science

^3^ Australian Institute of Marine Science

Correspondence to [Tiffany.Sih@my.jcu.edu.au](mailto:Tiffany.Sih@my.jcu.edu.au)

# Supplementary material: Video

Video legend:

Caught on camera: fishes of the Great Barrier Reef (Australia) sampled by Baited Remote Underwater Video Stations (BRUVS). Smaller and cryptic species would be under-sampled by other methods, such as fishing surveys, scientific trawling or fish traps. Video captured potential new fish species and new geographic records on reefs 54-260m deep. These deeper reefs at mesophotic and sub-mesophotic depths have never been surveyed by video before this study. This research was supported by AIMS@JCU, the Australian Institute of Marine Science and the ARC Centre of Excellence for Coral Reef Studies at James Cook University.
